# Complete Genome Sequences of Four *Bordetella pertussis* Vaccine Reference Strains from Serum Institute of India

**DOI:** 10.1128/genomeA.01404-16

**Published:** 2016-12-22

**Authors:** Michael R. Weigand, Yanhui Peng, Vladimir Loparev, Taccara Johnson, Phalasy Juieng, Sunil Gairola, Rakesh Kumar, Umesh Shaligram, Ramnath Gowrishankar, Hercules Moura, Jon Rees, David M. Schieltz, Yulanda Williamson, Adrian Woolfitt, John Barr, M. Lucia Tondella, Margaret M. Williams

**Affiliations:** a Centers for Disease Control and Prevention, Atlanta, Georgia, USA; b Serum Institute of India Pvt. Ltd., Pune, India

## Abstract

Serum Institute of India is among the world’s largest vaccine producers. Here, we report the complete genome sequences for four *Bordetella pertussis* strains used by Serum Institute of India in the production of whole-cell pertussis vaccines.

## GENOME ANNOUNCEMENT

Whooping cough (pertussis) is a contagious respiratory disease primarily caused by the Gram-negative bacterium *Bordetella pertussis*. Vaccination programs in many countries have successfully reduced disease incidence. Yet *B. pertussis* persists, and pertussis disease has resurged, in part due to genetic divergence of circulating strains. Although reference strains used for vaccine production vary (1), growing evidence suggests that *B. pertussis* is evolving under vaccine-driven selection (2–5). Here, we report the complete genome sequences of four strains (134, 509, 6229, 25525) used by Serum Institute of India to manufacture whole-cell pertussis vaccines.

Whole-genome shotgun sequencing was performed using a combination of the PacBio RSII (Pacific Biosciences, Menlo Park, CA, USA), Illumina MiSeq (Illumina, San Diego, CA, USA), and Argus (OpGen, Gaithersburg, MD, USA) platforms as described previously (6). Briefly, genomic DNA libraries were prepared for PacBio sequencing using the SMRTbell template prep kit version 1.0 and the polymerase binding kit P6 version 2; libraries for Illumina sequencing were prepared using the NEB ultra library prep kit (New England Biolabs, Ipswich, MA, USA). *De novo* genome assembly was performed using the Hierarchical Genome Assembly Process version 3 (Pacific Biosciences) at >140× coverage. The resulting consensus sequences were manually checked for circularity and reordered to match the start of Tohama I (CP010964) (6). To ensure accuracy, assemblies were confirmed by comparison to *KpnI* restriction digest optical maps using the Argus system (OpGen) with MapSolver version 2.1.1 (OpGen). For strains 6229 and 25525, putative repeat duplications identified by increased read coverage depth and optical map misalignment were resolved manually. Sequences were further “polished” by mapping Illumina MiSeq PE-300 reads using CLC Genomics Workbench version 9 (CLC bio, Boston, MA, USA). Final assemblies were annotated using NCBI’s Prokaryotic Genome Annotation Pipeline.

Isolate and assembly characteristics are summarized in Table 1. All four assemblies included the full complement of known (>40) *B. pertussis* virulence-associated genes. Assembled genomes varied in sequence and chromosomal structure, with 509 appearing similar to vaccine reference strain 10536 (CP012128) (7) and 134 matching a recent sequence of the same strain (CP016338) (7). Genomes of strains 6229 and 25525 were closely related and more similar to clinical isolates than to other vaccine reference strains when compared to available complete assemblies. Two genomes included direct duplication of an approximately 128-kb region flanked by copies of IS*481* that was present in two copies in 6229 and three copies in 25525 (Table 1). These duplications were not resolvable by sequencing alone, and proper assembly was achieved only with the aid of optical mapping. Gene content within this region was identical to Tohama I (BP1269 to BP1395, NC_002929) and encoded functions such as amino acid transport, stress responses, and flagellar biosynthesis.

**TABLE 1:** Characteristics of *B. pertussis* vaccine reference strains and genome assemblies

Strain	Genotype^a^	Genome size (bp)	CDSs^b^	Repeats^c^	Accession no.
134	*prn1-ptxP1-ptxA2*	4,128,984	3,645	NA^d^	CP017402
509	*prn7-ptxP2-ptxA4*	4,140,370	3,650	NA	CP017403
6229	*prn1-ptxP1-ptxA1*	4,257,407	3,767	1,324,103 to 1,452,037	CP017404
				1,453,081 to 1,581,015	
25525	*prn1-ptxP1-ptxA1*	4,386,396	3,882	1,324,106 to 1,452,040	CP017405
				1,453,084 to 1,581,018	
				1,582,062 to 1,709,996	

a All were *fimH1* and *ptxB2*.

b CDSs, coding sequences.

c Coordinates of direct repeats.

d NA, not applicable.

Multiple alignment of complete assemblies has shown that the *B. pertussis* genome exhibits considerable rearrangement plasticity (6, 8, 9) but has thus far not revealed large repeats like those in 6229 and 25525. However, duplication of genes within this same region was inferred by microarray hybridization in Finnish isolate KKK1330 (10). Homologous recombination between copies of IS*481* has contributed to genome reduction in *B. pertussis* (11) and these data suggest that expansion is also possible by the same mechanism.

### Accession number(s).

The complete genome sequences have been deposited at DDBJ/EMBL/GenBank under the accession numbers listed in Table 1. The versions described in this paper are the first versions.
